# Assessment of Serum Ferritin, Thyroid Profile and Calcium Homeostasis in Beta-Thalassemia Major Patients

**DOI:** 10.7759/cureus.104666

**Published:** 2026-03-04

**Authors:** Divya Gupta, Onkar Awadhiya, Ashok Kumar, Shiv R K Dubey, Jagdamba Dixit

**Affiliations:** 1 Department of Biochemistry, People's College of Medical Sciences and Research Centre (PCMS &amp; RC), Bhopal, IND; 2 Department of Internal Medicine, All India Institute of Medical Sciences (AIIMS), Nagpur, IND; 3 Department of Biochemistry, All India Institute of Medical Sciences (AIIMS), Bhopal, IND; 4 Department of Pediatrics, People’s College of Medical Sciences and Research Centre (PCMS &amp; RC), Bhopal, IND

**Keywords:** calcium homeostasis, serum ferritin, 𝛽-thalassemia major, thyroid profile, vitamin d

## Abstract

Background

𝛽-thalassemia major (𝛽-TM) is the most severe form of 𝛽-thalassemia. Serum ferritin is considered as a surrogate marker of iron overload and hemosiderosis. Iron deposition is the main cause of damage to endocrine glands in patients with 𝛽-TM. Furthermore, frequent blood transfusion (BT) may lead to altered calcium and phosphate homeostasis in patients. Increased ferritin levels and hepatic iron overload may be associated with vitamin D deficiency and altered bone metabolism. The aim of the study was to analyze serum ferritin, thyroid profile, vitamin D, serum calcium, and serum phosphate levels in 𝛽-TM patients undergoing BT. Further, the study aimed to determine the association of serum ferritin with thyroid profile, hematological parameters and calcium homeostasis in 𝛽-TM patients.

Materials & methods

𝛽-TM patients (N = 35) aged 6 months to 168 months were recruited. Laboratory investigations, CBC, serum ferritin, vitamin D, serum calcium, serum phosphate, and thyroid profile, were performed.

Statistical analysis

Statistical analysis was performed by SPSS version 21 (IBM Corp., Armonk, NY, USA). Data obtained from biochemical parameters - serum ferritin, vitamin D, serum calcium, serum phosphate, and thyroid profile from 𝛽-TM patients - were subjected to descriptive analysis. Normal distribution of variables was analyzed using the Shapiro-Wilk test. Data were not normally distributed; hence, nonparametric tests were used for statistical analysis. Bivariate correlation between two variables was analyzed by Spearman-rank’s correlation test.

Result

In our study, the mean serum ferritin value was 2328.6 ng/ml (159.60-5958.80), and 15 (42.8%) patients had severe hyperferritinemia. Female 𝛽-TM patients had severe vitamin D deficiency, and the frequency of hypocalcemia was higher in males and 4 (11%) patients showed deranged values of phosphate. 𝛽-TM patients had a deranged thyroid profile, 10 (28.5%) of the patients had low thyroxine (T4), whereas elevated TSH was noted in 15 (42.8%) patients.

Conclusion

Our study showed that hematological parameters such as WBC and platelet count exhibited association with serum vitamin D, T3, calcium and phosphate levels, but a large number of sample size is required to validate the findings. Routine monitoring of endocrine function or early detection of endocrine complications is needed to improve their quality of life. Our study generates preliminary data; further studies, multicentric studies or studies on larger sample sizes, are required.

## Introduction

𝛽-thalassemia major (𝛽-TM) is an autosomal recessive monogenic disorder. Globally, an estimated 300,000 to 400,000 babies are born with hemoglobinopathies, while approximately 23,000 babies are born with thalassemia major each year [1]. In India, 𝛽-thalassemia is prevalent across the country; around 10,000-12,000 𝛽-TM infants are born annually. Approximately 42 million 𝛽-thalassemia carriers are present with an average frequency of 3-4% [2, 3]. 𝛽-TM patients require lifelong repeated blood transfusions (BT) to maintain hemoglobin levels, which causes iron overload and hemosiderosis.

𝛽-TM patients have various growth and development issues, and it also affects multiple organs such as the liver, heart, and endocrine systems, thereby leading to poor morbidity and mortality [4, 5]. The management strategy of 𝛽-TM encompasses BT, hydroxyurea, chelation therapy for iron overload, splenectomy, stimulation of fetal Hb synthesis, and bone marrow transplantation [6]. Iron overload leads to a range of complications, including cardiac disease, pulmonary hypertension, liver disease, musculoskeletal disorder, chronic pain, and psychiatric disorders [5].

Endocrine glands are vulnerable to iron deposits; common endocrine complications of 𝛽-TM are diabetes mellitus, hypothyroidism, hypoparathyroidism, short stature, hypogonadotropic, and delayed puberty. In addition, complications may occur in other tissues and organ systems, including the heart, kidneys, bones, and eyes [1,7]. The benchmark test to assess tissue iron level is the tissue biopsy. However, serum ferritin is considered a surrogate iron overload marker (hemosiderosis). Besides bone rigidity, calcium and phosphate play a vital role in muscle function, nerve impulse transmission, intracellular signaling, and many glandular secretions. Iron overload due to frequent BT in 𝛽-TM patients may lead to altered calcium and phosphate homeostasis. Low vitamin D levels and altered bone metabolism have also been observed in 𝛽-TM patients. Regular monitoring of bone mineral density (BMD) and other biochemical parameters is advised for 𝛽-TM patients on regular BT [8].

Iron overload also affects hematological and immune parameters. Chronic transfusion, oxidative stress, and iron-mediated toxicity may influence leukocyte counts and immune competence, increasing susceptibility to infections and inflammatory complications. Exploring the association between serum ferritin and leukocyte parameters may therefore provide insights into iron-induced immune dysregulation in 𝛽-TM. Given the high prevalence of 𝛽-TM in India and the burden of transfusion-related complications, a comprehensive assessment of serum ferritin and its association with thyroid function, calcium homeostasis, and leukocyte parameters is essential. Such analysis may facilitate early identification of endocrine and metabolic abnormalities, enable timely therapeutic interventions, and improve long-term clinical outcomes and quality of life in transfusion-dependent 𝛽-TM children. Therefore, the aim of the study was to analyze serum ferritin, thyroid profile, vitamin D, serum calcium, and serum phosphate levels in 𝛽-TM patients undergoing BT. Further, the study aimed to determine the association between serum ferritin and thyroid profile, hematological parameters and calcium homeostasis in 𝛽-TM patients.

## Materials and methods

Patients and clinical examination

The hospital-based study was approved by the Institutional Ethics Committee of People’s College of Medical Sciences & Research (PCMS&RC), Bhanpur, Bhopal (Ref. no. PCMS/OD/PS/2023/993(9)). 𝛽-TM patients were recruited during July 2023-December 2023 at the Departments of Biochemistry and Pediatrics of the institute. 𝛽-TM patients aged 6 months to 14 years (6 months - 168 months), who underwent routine BT and received iron chelation treatment at the institute, were enrolled in the study. The duration of the study was six months, so a convenient and feasible sample size of 35 was chosen for a hospital-based pilot study.

All participants received a standard clinical examination, performed by a pediatrician. Diagnosis of 𝛽-TM was confirmed by hemoglobin electrophoresis. Clinical details were recorded from the patient’s data sheets (date of enrollment, date of and age at the time of diagnosis, details of hematological parameters, total number of BT; White Blood Cells (WBC)1-WBC at the time of diagnosis, WBC2-WBC at the time of previous BT, WBC3-WBC at the time enrollment, Platelet Count (PLT)1-PLT at the time of diagnosis, PLT2-PLT at the time of previous BT, PLT3-PLT at the time of enrollment, hemoglobin (Hb) value, height, weight, and pathological details as per the patient’s proforma). 𝛽-TM patients with the following criteria were excluded from the study: bone marrow transplanted patients; age less than six months or more than 14 years, or 168 months, during the study period; human immunodeficiency viruses (HIV)-infected patients; patients taking higher doses of steroids for two weeks or more; and patients with any other chronic illness such as tuberculosis, cancer, or any other inflammatory bone/joint disorder. The subjects were enrolled by consecutive sampling. Study samples were collected for laboratory investigations after obtaining informed consent from the parents, and verbal assent was also taken from the 𝛽-TM patients. Venous blood (08 ml) was drawn by venipuncture and distributed into two tubes: an ethylenediaminetetraacetic acid (EDTA) vacutainer and plain vacutainers.

Laboratory investigations

Methods to analyze various laboratory investigations and their reference values and cutoffs are shown in Appendix 1 and Appendix 2. Complete blood counts (CBC), hormone parameters, and clinical biochemistry parameters were performed using 5 parts Differential Hematology Analyzer (Vector Unicorn, Gujarat, India), MiniVidas Compact Multiparametric Immunoassay Analyzer (biomerieux, Marcy-l'Etoile, France), and BA200 biochemistry analyzer (BioSystems, Barcelona, Spain).

Statistical analysis

Clinico-pathological details of 𝛽-TM patients were entered into Microsoft Excel and exported to SPSS version 21 (IBM Corp., Armonk, NY, USA). Data obtained from biochemical parameters, i.e., serum ferritin, vitamin D, serum calcium, serum phosphate, and thyroid profile from 𝛽-TM patients, were subjected to descriptive analysis. Normal distribution of variables was analyzed using the Shapiro-Wilk test. Data were not normally distributed; hence, nonparametric tests were used for statistical analysis. Bivariate correlation between two variables was analyzed by Spearman’s rank correlation test.

## Results

A total of 35 𝛽-TM patients (22 males and 13 females), aged 6 months to 14 years (6 months-168 months), participated in the present study, and the calculated mean age was 86.17±47.63 months (13-156 months). Mean values of biochemical parameters and clinicopathological details of 𝛽-TM patients are shown in Table 1 and Table 2, respectively. Details of test investigations, methods, and reference ranges are shown in Appendices 1 and 2. Patients’ ages were categorized into three groups, i.e., Group I, Group II, and Group III.

**Table 1:** Mean values of all biochemical parameters of the 𝛽-TM patients TSH: thyroid-stimulating hormone; T3: thyronine; T4: thyroxine; BT: blood transfusion; 𝛽-TM: 𝛽-thalassemia major

Biochemical parameters	Mean Value	Range	
		Min.	Max.
Serum ferritin	2328.69	159.60	5958.80
T3 (nmol/L)	1.82	1.12	3.42
T4 (nmol/L)	95.10	64.04	129.05
TSH (µIU/L)	3.53	1.38	11.30
Vitamin D (ng/mL)	17.36	8.10	43.30
Serum Calcium (mg/dl)	9.0	7.69	11.57
Serum Phosphate (mg/dl)	5.63	11.57	13.19
Total no of BT	73.57	1.00	378.00

**Table 2:** Clinicopathological features of 35 𝛽-TM patients

Clinicopathological features	Number	Frequency (%)
Facial dismorphism	32	91.4
Splenomegaly	30	85.7
History of diabetes	00	0
Thyroid disorder	00	0
Hypothyroidism	00	0
Back pain	4	11.4
Bone pain	3	8.5
Pain in joints	7	20.0
Fracture	3	8.5
Kyphosis	00	0
Scoliosis	00	0
Lordosis	00	0
Genu Varum	00	0
Genu Valgum	00	0
Fatigability	23	65.7
Lethargy	29	82.8
Chest pain	6	17.0
Breathlessness at rest	20	57.1
Jaundice	7	20.0
Gall stone	1	2.8
Skin Pigmentation	00	0

Serum ferritin

All patients recruited in the study had iron overload as measured by serum ferritin levels. Out of 35 patients, 15 (42.8%), 12 (34.2%), and 8 (22.8%) exhibited severe, moderate, and mild iron overload, respectively, as assessed by serum ferritin levels. Categorization of serum ferritin data, based on patients’ age, showed that more than half of the patients (8, 57.1%) in group II had severely high serum ferritin levels, whereas moderately high levels (4, 50%) were observed in group III patients (Table 3).

**Table 3:** Serum ferritin levels in 𝛽-TM patients ^§^ Group I, 6-60 months; Group II, 61-120 months; Group III, 121-156 months; M, Male; F, Female * Source: Ref. [1].

Serum Ferritin (ng/ml)		Mild <1000	Moderate 1000 - <2000	Severe >2000
		08 (22.8%)	12 (34.2%)	15 (42.8%)
Gender	Male (N=22)	5 (22.7%)	6 (27.2%)	11 (50%)
	Female (N=13)	3 (23.0%)	6 (46.1%)	4 (30.7%)
Age (Months)§	Group I, 13 (9M/4F)	05 (38.4%)	04 (30.7%)	04 (30.7%)
	Group II, 14 (9M/5F)	02 (14.2%)	04 (28.5%)	08 (57.1%)
	Group III, 8 (4M/4F)	01 (12.5%)	04 (50%)	03 (37.5%)

Serum vitamin D

To ascertain 𝛽-TM patients’ calcium homeostasis and bone health, we measured serum vitamin D, serum calcium, and serum phosphate. Most of the patients (88.6%) had low levels of vitamin D, and only 4 (11.4%) patients had normal levels. We categorized patients according to vitamin D levels: severe deficiency level (<10 ng/ml), deficiency (10-20 ng/ml), insufficiency (20-30 ng/ml), and sufficiency level (>30 ng/ml). The majority of the patients, 20 (57.1%), had vitamin D deficiency, and 7 (20%) had severe vitamin D deficiency. A higher proportion of female patients had severe vitamin D deficiency compared to male patients, 4 (30.7%) versus 3 (13.6%). Severe deficiency of vitamin D was common in Group III, as shown in Table 4.

**Table 4:** Vitamin D levels in the 𝛽-TM patients §Group I, 6-60 months; Group II, 61-120 months; Group III, 121-156 months; M, Male; F, Female

Vitamin D (ng/mL)		Severe Deficiency <10	Deficiency 10-20	Insufficiency 20-30	Sufficiency (Normal) >30
		7 (20%)	20 (57.1%)	4 (11.4%)	4 (11.4%)
Gender	Male (N=22)	3 (13.6%)	12 (54.5%)	4 (18.1%)	3 (13.6%)
	Female (N=13)	4 (30.7%)	8 (61.5%)	-	1 (7.6%)
Age§ (Months)	Group I, 13 (9M/4F)	1 (7.6%)	8 (61.5%)	2 (15.3%)	2 (15.3%)
	Group II, 14 (9M/5F)	3 (21.4%)	8 (57.1%)	2 (14.2%)	1 (7.1%)
	Group III, 8 (4M/4F)	3 (37.5%)	4 (50%)	-	1 (12.5%)

Serum calcium and phosphate

Approximately 17 (48.5%) 𝛽-TM patients had hypocalcemia, and 1 (2.8%) patient exhibited hypercalcemia. The frequency of hypocalcemia was higher in male 𝛽-TM patients compared to females. Approximately 4 (11%) of patients showed deranged values of phosphate: hypophosphatemia 2 (5.7%) and hyperphosphatemia 2 (5.7%) (Table 5).

**Table 5:** Serum calcium and serum phosphate in 𝛽-TM patients § Group I, 6-60 months; Group II, 61-120 months; Group III, 121-156 months

Calcium (mg/dl)		Normal	Low	High
		17 (48.5%)	17 (48.5%)	1 (2.8%)
Gender	Male (N=22)	9 (40.9%)	12 (54.5%)	1 (4.5%)
	Female (N=13)	8 (61.5%)	5 (38.4%)	-
Age§ (Months)	Group I 13 (9M/4F)	6 (46.1%)	7 (53.8%)	-
	Group II 14 (9M/5F)	6 (42.8%)	7 (50%)	1 (7.1%)
	Group III 8 (4M/4F)	5 (62.5%)	3 (37.5%)	-
Phosphate (mg/dl)		Normal	Low	High
		31 (88.5%)	2 (5.7%)	2 (5.7%)
Gender	Male (N=22)	19 (86.3%)	2 (9.0%)	1 (4.5%)
	Female (N=13)	12 (92.3%)	-	1 (7.6%)
Age (Months) §	Group I, 13 (9M/4F)	11 (84.6%)	1 (7.6%)	1 (7.6%)
	Group II, 14 (9M/5F)	13 (92.8%)	1 (7.1%)	-
	Group III, 8 (4M/4F)	7 (87.5%)	-	1 (12.5%)

Thyroid profile

Thyroid dysfunction is a very common complication in 𝛽-TM patients. Based on total thyronine (T3), thyroxine (T4), and thyroid stimulating hormone (TSH) levels, patients were categorized as primary hypothyroidism (T3 - low/normal, T4 - low, TSH - high), hypothyroxinemia (T3 - low/normal, T4 - low, TSH - normal) and subclinical hypothyroidism (T3 - normal, T4 - normal, TSH - high) as shown in the table in Appendix 3. Thyronine (T3) levels were in the normal range in patients; however, 10 (28.5%) of the patients had low thyroxine (T4). As shown in Table 6 and Table 7, elevated TSH was reported in 8 (22.8%) - 3 (37.5%), 4 (28.5%), and 1 (7.6%) in groups III, II, and I, respectively. Clinical interpretation of the thyroid profile showed that 1 (2.8%), 9 (25.7%), and 7 (20%) patients had primary hypothyroidism, hypothyroxinemia, and subclinical hypothyroidism, respectively. Older patients showed an increased frequency of subclinical hypothyroidism compared to younger patients, and mostly hypothyroxinemia was noted in group I (5, 38.4%) and group II (4, 28.5%) patients, as shown in Table 6 and Table 7.

**Table 6:** Thyroid profile (T3, T4, TSH) in 𝛽-TM patients ^§^ Group I, 6-60 months; Group II, 61-120 months; Group III, 121-156 months

T3 (nmol/L)		Normal	Low	High
		35 (100%)	-	-
Gender	Male (N=22)	22 (100%)	-	-
	Female (N=13)	13 (100%)	-	-
Age (Months)§	Group I, 13 (9M/4F)	13 (100%)	-	-
	Group II, 14 (9M/5F)	14 (100%)	-	-
	Group III, 8 (4M/4F)	8 (100%)	-	-
T4 (nmol/L)		25 (71.4%)	10 (28.5%)	-
Gender	Male (N=22)	16 (72.7%)	6 (27.2%)	-
	Female (N=13)	9 (69.2%)	4 (30.7%)	-
Age (Months) §	Group I, 13 (9M/4F)	8 (61.5%)	5 (38.4%)	-
	Group II, 14 (9M/5F)	9 (64.2%)	5 (35.7%)	-
	Group III, 8 (4M/4F)	8 (100%)	-	-
TSH (mIU/L)		27 (77.1%)	-	8 (22.8%)
Gender	Male (N=22)	16 (72.7%)	-	6 (27.2%)
	Female (N=13)	11 (84.6%)	-	2 (15.3%)
Age (Months) §	Group I, 13 (9M/4F)	12 (92.3%)	-	1 (7.6%)
	Group II, 14 (9M/5F)	10 (71.4%)	-	4 (28.5%)
	Group III, 8 (4M/4F)	5 (62.5%)	-	3 (37.5%)

**Table 7:** Clinical interpretation of thyroid profile (T3, T4, TSH) in 𝛽-TM patients § Group I, 6-60 months; Group II, 61-120 months; Group III, 121-156 months

Age (Months)§	N (%)	Group I, 13 (9M/4F)	Group II, 14 (9M/5F)	Group III, 8 (4M/4F)
Normal	18 (51.4%)	7 (53.8%)	6 (42.8%)	5 (62.5%)
Primary hypothyroidism	1 (2.8%)	-	1 (7.14%)	-
Hypothyroxinemia	9 (25.7%)	5 (38.4%)	4 (28.5%)	-
Subclinical hypothyroidism	7 (20%)	1 (7.6%)	3 (21.4%)	3 (37.5%)

Bivariate correlation analysis

As shown in Table 8, correlations between the parameters such as T3, T4, serum phosphate, vitamin D, serum ferritin, PLT3, PLT2, PLT1, WBC1, WBC2, and WBC3 were analyzed by bivariate correlation analysis. Serum vitamin D levels were strongly correlated with WBC, serum ferritin with the total number of BT, T3 with serum phosphate, and T4. A moderate association was observed for serum phosphate with PLT2 and T4 with WBC3, as shown in Table 8.

**Table 8:** Bivariate correlation among the parameters *WBC1: Patient WBC value at the time of diagnosis; WBC2: Patient WBC value last visited for blood transfusion (BT); WBC3: Patient WBC value at the time of study recruitment. *PLT2: Patient PLT value last visited for the blood transfusion (BT).

Parameters	Correlation	Pearson Correlation	Sig. (2-tailed)
T3	Phosphate	0.488**	0.003
T3	T4	0.407*	0.015
T4	WBC3	0.377*	0.026
Vitamin D	WBC1	0.502**	0.002
Vitamin D	WBC2	0.441**	0.008
Vitamin D	WBC3	0.417*	0.013
Ferritin	Total BT number	0.525**	0.001
Phosphate	PLT2	0.369*	0.029
Non-parametric Spearman-rank correlation test			
Parameters	Correlation	Correlation Coefficient (rho/r)	Sig. (2-tailed)
T3	Phosphate	0.531**	0.001
T3	T4	0.353*	0.038
T3	WBC2	0.498**	0.002
Calcium	WBC1	0.467**	0.005
Vitamin D	WBC2	0.370*	0.029
Vitamin D	WBC3	0.419*	0.012
Ferritin	Total BT number	0.536**	0.001
Total BT number	WBC2	-0.380*	0.024
Total BT number	WBC3	-0.407*	0.015
WBC1	WBC3	0.361*	0.033
WBC2	WBC3	0.575**	0.000
WBC3	PLT3	0.449**	0.007

In our study, serum total T3 levels were strongly correlated with serum phosphate (r = 0.531, p < 0.001) and WBC2 (r = 0.498, p < 0.002) in 𝛽-TM patients. Total T3 levels were also moderately correlated with serum T4 levels (r = 0.353, p < 0.038). Ferritin levels were strongly correlated (r = 0.536, p < 0.001) with the total BT number in 𝛽-TM patients. Serum vitamin D levels showed moderate to strong correlation with WBC2 and WBC3 (r = 0.370, p < 0.029) and (r = 0.419, p < 0.012), respectively. Serum calcium value also showed a strong association with WBC1 (r = 0.467, p < 0.005). On the other hand, the total number of BT showed a moderate negative correlation with WBC2 and WBC3 (r = -0.380, p < 0.024; r = -0.407, p < 0.015, respectively), as shown in Table 8.

## Discussion

It is a well-known fact that the number of BT is a significant cause of hepatic iron overload in transfusion-dependent 𝛽-TM patients, and iron chelation therapy leads to significant improvement for 𝛽-TM patients. The development of bone diseases associated with thalassemia is influenced by a complex interplay of factors. These include hormonal deficiencies, particularly hypogonadism and hypoparathyroidism, which are often secondary to hemosiderosis. Other contributing factors include marrow expansion, iron overload, and toxic effects of iron chelation therapy, all of which significantly disrupt bone metabolism [9]. Vitamin D deficiency can result in decreased bone mineralization and loss of cortical bone, leading to conditions such as osteopenia, osteoporosis, scoliosis, skeletal deformities, bone pain, nerve compression, and an increased risk of spontaneous pathological fractures [10].

In the current study, we measured serum calcium, serum phosphate and vitamin D levels in 𝛽-TM patients and correlated them with serum ferritin and thyroid profile. Hypoparathyroidism is a primary condition that can lead to hypocalcemia and osteoporosis. Intestinal calcium absorption is regulated by vitamin D, which directly stimulates calcium absorption by upregulating the expression and activities of several calcium transporters, such as Transient Receptor Potential Cation Channel Subfamily V Member 5 (TRPV5) and Transient Receptor Potential Vanilloid Member 6 (TRPV6) and calbindinD9k (CaBP-9k) [11,12].

Sultan et al. reported hypocalcaemia and hypophosphatemia in 66.6% and 19.4% 𝛽-TM patients, respectively [13], whereas in our study, 48.5% and 2.8% patients had hypocalcemia and hypercalcemia, respectively. Altered serum calcium and phosphate levels have been found in previous studies, with a prevalence of 4-40%, and a higher preponderance in males. We have reported significant hypocalcemia (48.5%), and our findings are also supported by the previous findings [13,14]. In contrast, some authors did not find a significant difference in serum calcium levels between 𝛽-TM patients and controls [15,16].

Iron chelation therapy may also contribute to hypocalcemia [17]. In our study, patients had a low frequency of hypophosphatemia (5.7%) and hyperphosphatemia (5.7%), and our findings corroborate with previous studies [13,18]. El-Nashar et al. reported that an altered calcium-phosphorus ratio in 𝛽-TM patients with osteoporosis may be a predisposing factor for bone involvement [9].

A study reported vitamin D deficiency in 72.2% of 𝛽-TM patients [14]. In contrast, our study showed that serum vitamin D levels were low in 89% (severe deficiency, deficiency, insufficiency) of 𝛽-TM patients. Mostly, vitamin D deficiency was observed in age group II (61-120 months). A study from India compared vitamin D levels in thalassemia patients with age-matched controls and found low levels of vitamin D in the patients’ group (13.12±2.90 vs 24.2±4.1 ng/ml) [19].

Low levels of vitamin D may be due to malabsorption, insufficient dietary intake of vitamin D, or defective hydroxylation of vitamin D (hepatic dysfunctions) [20]. A recent article reported that deposition of iron in the liver and skin disrupts hydroxylation and vitamin D synthesis; therefore, 𝛽-TM patients might have vitamin D deficiency [21]. Thus, iron overload associated with frequent BT may lead to vitamin D deficiency, which might be due to hepatic iron overload rather than dysfunctioning endocrine systems [22].

Napoli et al. had reported a significant negative correlation of vitamin D with age and serum ferritin level (r = −0.28, p < 0.05; r = −0.31, p < 0.05), respectively [23]. In our study, WBCs have a strong correlation with vitamin D, which shows that low WBC indicates vitamin D deficiency in patients. Vitamin D is known to boost the immune system, as vitamin D receptors are present in WBCs. Supplements of vitamin D and calcium are recommended to prevent osteopenia and severe bone diseases for 𝛽-TM patients.

High serum ferritin level is associated with increased endocrine disorders and cardiovascular disease, whereas chelation therapy decreases the prevalence of endocrinopathies. Several studies from India showed huge variations in mean serum ferritin levels 557.2±198.6, 1296.6±1009.6, and 2903.10±772.2 reported by Jaipuria et al., Khandelwal et al., and Kundu et al., respectively [24-26]. In our study, the mean value of serum ferritin was 2328.6 with a range of 159.6-5958.8, and 42.8% patients had a severe increase in serum ferritin levels. Serum ferritin value is directly proportional to the number of BT or vice versa. In order to reduce levels of hepatic iron accumulation, iron chelators (FDA-approved chelators - Deferoxamine (DFO), Deferiprone (DFP), and Deferasirox (DFX), Desiferox) are prescribed to 𝛽-TM patients as per their standard guidelines and management [27]. In our study, approximately 94.2% (33/35) of patients received regular iron chelation therapy and also showed a strong correlation with the number of BT (r = 0.536 and p < 0.001). A cross-sectional study of 58 patients from Indonesia showed a mean serum ferritin of 5,340 with a range of 355-22,352 [28]. Singhal and Goyal showed that the patients <10 years of age had ferritin levels ≤2000 ng/ml, whereas patients >10 years of age had ferritin levels >2000 ng/ml [29]. As per the standard guideline by Cappellini et al., the concentration of serum ferritin should be measured every three months (1-3 months). Currently, the target value of serum ferritin in 𝛽-TM patients is in the range of 500 and 1000 μg/L; thus, serum ferritin measurement is a more reliable indicator of iron overload [27].

Our study found a significant correlation of T3 with serum phosphate, T4, and WBC3. Khandelwal et al. reported overt hypothyroidism (6.8%) and subclinical hypothyroidism (13.6%) in 𝛽-TM patients [25]; however, primary hypothyroidism (2.8%), hypothyroxinemia (25.7%), and subclinical hypothyroidism (20%) were observed in our study. The frequency of subclinical hypothyroidism and hypothyroxinemia was higher in the current study compared to a previous study [25]. Khandelwal et al. also reported a negative correlation between serum ferritin and TSH. The mean serum ferritin levels in Khandelwal’s study were 1296; however, in our study mean serum ferritin levels were 2328.6. Thus, the high occurrence of subclinical hypothyroidism and hypothyroxinemia could be explained by higher levels of serum ferritin in the patients enrolled in our study.

Singhal and Goyal reported thyroid dysfunction in 26.6% in 𝛽-TM patients, with hypothyroidism and subclinical hypothyroidism observed in 19% and 8.6% of participants, respectively [29]. Another study showed hypothyroidism in 23.3% of patients, and reported that compensated hypothyroidism (normal T3 and T4 with increased TSH) was found in 15% of patients, and decompensated hypothyroidism was found in 8.33% of patients [24]. Hypothyroidism and subclinical hypothyroidism in patients may be due to damage of thyroid gland caused by iron overload and the release of free radicals. The degree of iron overload is directly related to the incidence of hypothyroidism. Agarwal et al. showed that thyroid abnormality was among 19.4% 𝛽-TM patients; chronic hypoxia and iron overload are postulated to cause damage to the thyroid gland [30].

We also demonstrate that the hematological parameters WBC1, WBC2 and WBC3 correlate with serum vitamin D, T3, calcium, number of BT, and platelet counts. None of the previously published studies has reported such an association in 𝛽-TM patients. The association needs to be further validated in other studies with a large sample size. Elevated serum ferritin levels suggest that frequent monitoring of serum ferritin levels is very crucial. At the time of enrollment of patients and data acquisition for our study, we also observed that there was a non-compliance with monitoring serum ferritin levels in patients. Testing of serum ferritin is not covered in government facilities. Most of the patients belonged to the lower socioeconomic conditions and were unable to bear the cost of serum ferritin testing. This is one of the major causes of the increased severity of iron overload, causing endocrinopathy and hepatic damage.

Limitation of the study

However, the present study has certain limitations, including a relatively small sample size (35 𝛽-TM patients). Due to funding limitations, we could not perform PTH test. Serum ferritin testing is not available at government-funded primary health centers and represents a financial burden for patients. Higher numbers of study samples are needed to validate the result to improve quality and prevent complications in 𝛽-TM patients.

## Conclusions

𝛽-TM patients have a markedly deranged biochemical profile. Regular monitoring of serum ferritin, BMD, thyroid profile and other biochemical parameters will improve bone health in 𝛽-TM patients. In our study, most of the deranged values of biochemical parameters were found in patients older than 5 years. Our study shows that hematological factors such as WBC and platelet count might play a significant role in indicating serum vitamin D, T3, calcium and phosphate levels, but a large sample size is required to validate the findings. Prevention of vitamin D deficiency is helpful to improve bone mineralization and potentially prevent osteoporotic fracture. Calcium and vitamin D supplementation is commonly required; therefore, comprehensive nutritional support and appropriate supplementation are strongly recommended for patients with 𝛽-TM. In our study, approximately 49% of 𝛽-TM patients clinically showed abnormal thyroid profile. Therefore, thalassemia patients need routine monitoring of endocrine function or early detection of endocrine complications to improve their quality of life.

## Appendices

Appendix 1

**Table 9:** Details of laboratory investigation, method, kit and machine

Investigation		Method	Kit	Machine
Blood profile	CBC	Flow Cytometry (FCM)+ Laser scatter + Chemical dye method for WBC differential analysis and WBC counting	Auto hematology analyzer	Vector Unicorn 5 part Differential Hematology Analyzer
Serum Ferritin	Serum Ferritin	ELFA (Enzyme-Linked Fluorescent Assay)	Ref 30411 VIDAS® FER	Biomerieux MiniVidas Compact Multiparametric Immunoanalyzer
Thyroid profile	Triiodothyronine (T3)		Ref 30402 VIDAS® T3	Biomerieux MiniVidas Compact Multiparametric Immunoanalyzer
	Thyroxine (T4)		Ref 30404-01 VIDAS® T4	Biomerieux MiniVidas Compact Multiparametric Immunoanalyzer
	Thyroid-stimulating hormone (TSH)		Ref 30400-01 VIDAS® TSH	Biomerieux MiniVidas Compact Multiparametric Immunoanalyzer
Calcium, phosphate and vitamin D	Vitamin D		Ref 30463 VIDAS 25 OH Vitamin D TOTAL	Biomerieux MiniVidas Compact Multiparametric Immunoanalyzer
	Serum Calcium	Arsenazo	112	BioSystems BA200 biochemistry analyzer
	Serum Phosphate	Phosphomolybdate	54901	BioSystems BA200 biochemistry analyzer

Appendix 2

**Table 10:** Reference range of the biochemical parameters used in the study

S. No.	Parameters	Ref. value with unit	Interpretation	References
1.	Calcium (mg/dl)	8.8-10.8 (mg/dl)	8.8-10.8-Normal	[31]
			<8.8-Low	
			>10.8-High	
2.	Phosphorus (mg%)	4.0-7 (mg%)	4-7-Normal	[31]
			<3.9-Low	
			>7.1-High	
3.	Ferritin (ng/ml)	22-322 ng/ml (males); 10-291 ng/ml (females)	Mild <1000	[1]
			Moderate >1000	
			Severe >2000	
4.	Vit D (ng/ml)	30-80 (ng/ml)	Severe Deficient <10	[32]
			Deficiency 10-20	
			Insufficiency 20-30	
			Sufficiency >30	
5.	T3 (nmol/L)	1-5 yrs, 1.45-4.14 (nmol/L)	[31]	
		5-10 yrs, 1.26-3.71 (nmol/L)		
		10-14 yrs, 1.26-3.28 (nmol/L)		
6.	T4 (nmol/L)	1-5 yrs, 94-194 (nmol/L)	[31]	
		5-10 yrs, 83-172 (nmol/L)		
		10-14 yrs, 72-151 (nmol/L)		
7.	TSH (uIU/ml)	1-5 yrs, 0.7-6 (uIU/ml)	[31]	
		5-10 yrs, 0.6-4.8 (uIU/ml)		
		10-14 yrs, 0.5-4.3 (uIU/ml)		

Appendix 3

**Table 11:** Clinical Interpretation of Thyroid Profile

Thyronine (T3)	Thyroxine (T4)	Thyroid stimulating hormone (TSH)	Clinical Interpretation
Low/Normal	Low	High	Primary Hypothyroidism
Low/Normal	Low	Normal	Hypothyroxinemia
Normal	Normal	High	Subclinical Hypothyroidism
